# Exploring host-pathogen interactions through genome wide protein microarray analysis

**DOI:** 10.1038/srep27996

**Published:** 2016-06-15

**Authors:** Luigi Scietti, Katia Sampieri, Irene Pinzuti, Erika Bartolini, Barbara Benucci, Alessia Liguori, Andreas F. Haag, Paola Lo Surdo, Werner Pansegrau, Vincenzo Nardi-Dei, Laura Santini, Seguinde Arora, Xavier Leber, Simonetta Rindi, Silvana Savino, Paolo Costantino, Domenico Maione, Marcello Merola, Pietro Speziale, Matthew J. Bottomley, Fabio Bagnoli, Vega Masignani, Mariagrazia Pizza, Meike Scharenberg, Jean-Marc Schlaeppi, Mikkel Nissum, Sabrina Liberatori

**Affiliations:** 1 GSK Vaccines, Via Fiorentina 1, 53100 Siena, Italy; 2 Novartis Institutes for Biomedical Research, Novartis Campus, 4056 Basel, Switzerland; 3 Department of Molecular Medicine, Unit of Biochemistry, Viale Taramelli 3/b, 27100 Pavia, Italy; 4 University of Naples “Federico II”, Department of Biology, via Cinthia 4, 80126 Naples, Italy

## Abstract

During bacterial pathogenesis extensive contacts between the human and the bacterial extracellular proteomes take place. The identification of novel host-pathogen interactions by standard methods using a case-by-case approach is laborious and time consuming. To overcome this limitation, we took advantage of large libraries of human and bacterial recombinant proteins. We applied a large-scale protein microarray-based screening on two important human pathogens using two different approaches: (I) 75 human extracellular proteins were tested on 159 spotted *Staphylococcus aureus* recombinant proteins and (II) *Neisseria meningitidis* adhesin (NadA), an important vaccine component against serogroup B meningococcus, was screened against ≈2300 spotted human recombinant proteins. The approach presented here allowed the identification of the interaction between the *S. aureus* immune evasion protein FLIPr (formyl-peptide receptor like-1 inhibitory protein) and the human complement component C1q, key players of the offense-defense fighting; and of the interaction between meningococcal NadA and human LOX-1 (low-density oxidized lipoprotein receptor), an endothelial receptor. The novel interactions between bacterial and human extracellular proteins here presented might provide a better understanding of the molecular events underlying *S. aureus* and *N. meningitidis* pathogenesis.

Protein-protein interactions (PPIs) play a fundamental role in initiating and sustaining bacterial infections in the human body. PPIs are key to penetration of host barriers, from colonization of mucosal epithelia to invasion of host cells and tissues, as well as to evasion of host innate and adaptive immune responses^1^. Despite the biological relevance of PPIs at the host-pathogen interface, their systematic characterization is still challenging.

Microarrays represent a powerful tool for large-scale screenings, and this technology has been successfully applied to the identification of novel PPIs in different organisms. However, only a few examples exist where interactions between extracellular proteins from human and pathogen libraries were tested. The highest throughput was achieved by Wright and collaborators in studies reporting the systematic screen for interactions involved in the recognition of the host erythrocyte by the blood stage of the malaria parasite, where 40 human erythrocyte receptors were screened against 35 *Plasmodium falciparum* extracellular proteins and led to the identification of two novel erythrocyte receptors for *P. falciparum* parasites^2^,^3^,^4^. Similar studies were carried out to identify bacterial/human interactions, but involved a very limited number of human proteins^5^,^6^.

A large collection of human recombinant proteins exists at the Genomic Institute of the Novartis Research Foundation (GNF)^7^. In its current version, the GNF library consists of ≈2300 distinct proteins that have been prioritized from approximately 3500 human genes *in silico* predicted to code for secreted or single-pass transmembrane proteins. Such a large collection of recombinant human extracellular proteins represents a rich source of targets for bacterial effectors. In the present work, we describe a large-scale screening of such a library against relevant bacterial proteins of two important pathogens, *i.e. Staphylococcus aureus* and Serogroup B *Neisseria meningitidis* (meningococcus B, *N. meningitidis* group B) to identify new interactions.

*S. aureus* is a gram-positive bacterium and opportunistic pathogen living as a commensal in human skin and nasal cavities in 20% of the human population^8^. Several human proteins are targeted by *S. aureus* extracellular proteins^9^. In recent years it also became evident that staphylococcal evasion molecules may have multiple targets^10^. This suggests a complex network of interactions between *S. aureus* and the human extracellular proteome, providing the rationale for further investigations at the host-pathogen interface.

*N. meningitidis* group B is a Gram-negative encapsulated bacterium and commensal of human nasopharynx, which can become an aggressive pathogen leading to fulminant sepsis and meningitis. Recently, a four component protein-based vaccine (Bexsero^®^) was licensed by Novartis vaccines (now a GSK company). The Bexsero formulation contains the Neisserial adhesin A (NadA) which constitutes a key determinant of the vaccine-induced immunity^11^. NadA is a trimeric coiled-coil outer membrane protein constituted by an N-terminal “head” domain, a coiled-coil “stalk” and a transmembrane domain that anchors the protein to the bacterial membrane^12^. The gene is present in three out of four known hyper virulent lineages of *N. meningitidis* group B strains and several studies already demonstrated its importance during bacterial pathogenesis^13^,^14^,^15^. In addition, the crystal structure of a soluble ectodomain fragment of NadA variant 5 was recently solved^16^. Nevertheless, a global picture of the NadA interactions with the human extracellular proteome is still missing and might help in the understanding of *N. meningitidis* group B pathogenesis.

To our knowledge, we report here the largest microarray screening carried out so far between human and bacterial extracellular proteins using two different approaches. The *S. aureus* extracellular proteome was screened against a selection of human complement factors and extracellular matrix proteins, and led to the identification of the human complement factor C1q as a new target for the well-known staphylococcal immune evasion protein FLIPr. In a second experimental set-up, the complete library consisting of 2354 human extracellular proteins was screened to identify novel human receptors for NadA, and the oxidized low-density lipoprotein receptor LOX-1 was identified as the first putative endothelial receptor for this important neisserial adhesin.

## Results

### Two different microarray-based set-ups were applied to the discovery of novel host-pathogen interactions

**Figure 1:**
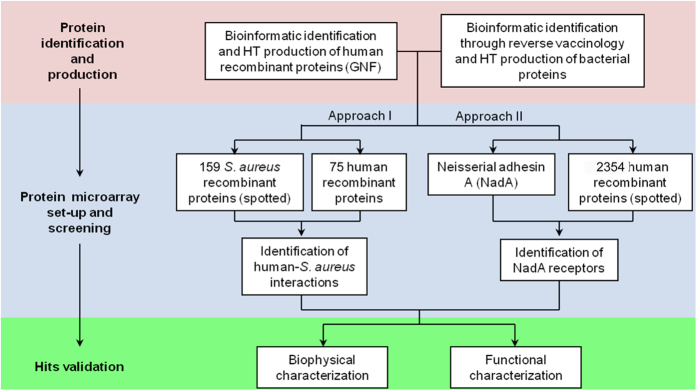
Conceptual organization of the workflow. We divided the work in three main steps: the protein identification and production, the protein microarray preparation and screening, and the hits validation. The two different approaches applied to *S. aureus* and to *N. meningitidis* are shown.

The overall strategy for the microarray-based identification of new interactions between human and bacterial extracellular proteins is reported in Fig. 1. Two different microarray screening setups were designed for the two pathogens, trying to answer different biological questions. The first setup had the primary objective of acquiring a picture of the reciprocal interactions between staphylococcal and human extracellular proteins. A complete unbiased approach in this setup would have allowed the screening of 381,600 different interactions, i.e. 159 staphylococcal proteins tested against 2400 human proteins. To show the feasibility of the approach, a subset of the human library consisting of major components from the complement system and extracellular matrix was selected for expression and purification, since these protein classes represent the first line of interaction at the host-pathogen interface. The resulting 75 recombinant proteins were tested against the staphylococcal library, leading to the remarkable number of 11,925 different interactions screened. The second set-up had the main goal of identifying new soluble/cellular receptors for the meningococcal adhesin NadA. In an unbiased approach, the complete library was expressed and NadA screened against it.

### Protein microarray led to the identification of 17 interactions with high Mean Fluorescence Intensity (MFI) between *S. aureus* and human proteins

**Figure 2:**
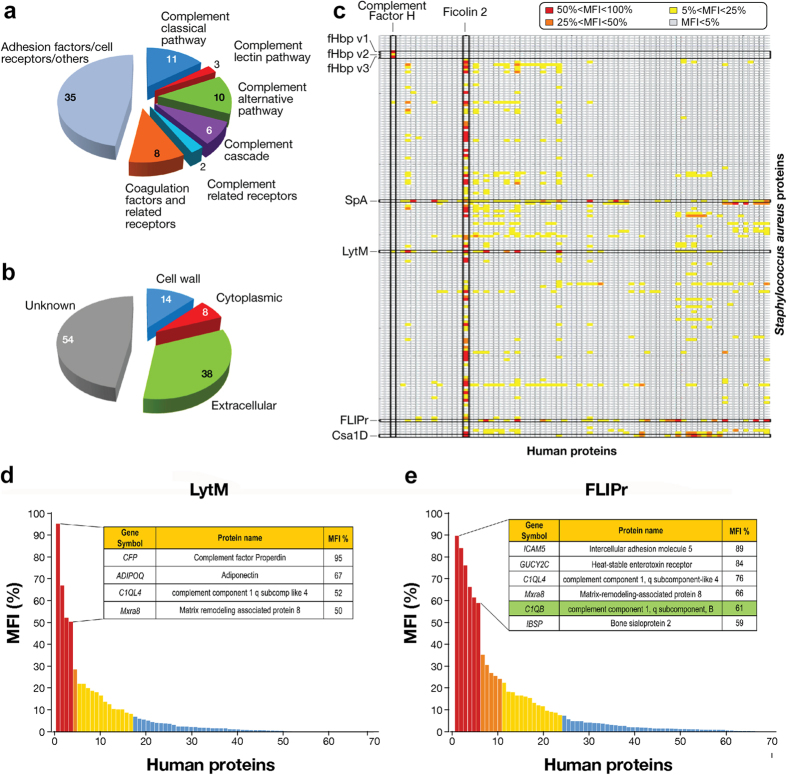
Protein microarray applied to *S. aureus*: design and results. (**a)** Classification of the 75 human proteins tested in the microarray screening based on their biological function and **(b)** of the *S. aureus* proteins spotted on the chip based on their predicted localization. **(c)** Schematic overview of the grid resulting from the screening. *S. aureus* proteins (lines) spotted on the chip were plotted against human proteins (columns) tested in overlay. Cells contain MFI value for each pair. Color code is used for visual information and to identify the three MFI cut-off thresholds (grey: MFI < 5%; yellow: 5% < MFI < 25%; orange: 25% < MFI < 50%; red: 50% < MFI < 100%. Human complement factor H and ficolin-2 and the bacterial fHbp v 1, 2 and 3 (neisserial proteins used as control), SpA, FLIPr and Csa1D are highlighted. **(d)** Plotting of MFIs of the 75 human proteins on the spotted LytM. Inset table shows MFI values, protein name and gene symbol of the 4 human proteins displaying fluorescence intensities above 50% MFI. **(e)** Plotting of MFIs of the 75 human proteins on the spotted FLIPr. Inset table shows MFI values, protein name and gene symbol of the first 20 human proteins. C1qB subcomponent is highlighted in green.

The protein microarray screening was performed testing 159 purified recombinant *S. aureus* proteins spotted onto nitrocellulose coated glass slides against 75 human recombinant proteins that include human complement factors and extracellular matrix proteins (Fig. 2a and Supplementary Table S1). The *S. aureus* proteins printed on the arrays represent surface and secreted factors selected *in silico* using a combined bioinformatics approach^17^,^18^. A total of 114 unique proteins were spotted, of which 104 were included as single constructs and 10 as multiple constructs for a total of 159 recombinant constructs (Fig. 2b and Supplementary Table S2). Supplementary Fig. S1 and supplementary Table S3 show the results obtained; out of 11766 possible binary interactions, 11168 (94.92%) resulted below the background threshold and were thereby considered as negative, while 598 (5.08%) resulted positive. Among these, 518/598 (4.40%) were ranked as low, 61/598 (0.52%) as medium and 19/598 (0.16%) as highly reactive (Supplementary Fig. S1, upper left panel) based on their MFI signals. Within the top 19 interactions (Supplementary Table S4), factor H binding protein (fHbp) variants 1 and 3 - which constituted the positive controls - were found when tested with human complement factor H. Among the tested human proteins, ficolin-2 showed medium/high binding signals with 80 out of the 159 bacterial proteins tested (Fig. 2c). Although we cannot exclude that a specific binding may occur between ficolin-2 and some staphylococcal proteins, this result seems to suggest that components other than the specific proteins spotted on the chip are recognized. In fact, ficolin-2 is known to bind to N-acetylglucosamine (GlcNAc) sugar residues^19^ and our analysis may have been compromised in this case by the presence of residual GlcNAc-containing LPS in the staphylococcal recombinant proteins produced in *E. coli*. Similarly, since the detection system relies on the use of antibodies and several human proteins were fused to an Fc-tag, the putative hits of the staphylococcal protein A (SpA) were not considered for further validation, knowing its ability to bind immunoglobulins. The rest of the highly reactive hits were shared between the staphylococcal glycyl-glycine endopeptidase LytM (4 hits), the conserved staphylococcal antigen 1D (Csa1D) (1 hit), and the FPRL1 (formyl peptide receptor-like 1) inhibitory protein (FLIPr) (6 hits). LytM (Fig. 2d) showed high reactivity signals with the complement factor P (CFP) (MFI score of 95%), adiponectin protein (ADIPOQ) (MFI score of 67%), the complement component 1q subcomponent like 4 (C1QL4) (MFI score of 52%) and the matrix remodelling associated protein 8 (MXRA8) (MFI score of 50%). Csa1D was instead highly reactive with ficolin-1 (FCN1). FLIPr showed several interesting putative interactors (Fig. 2e): the intercellular adhesion molecule 5 (ICAM5) (MFI score of 89%), the heat stable enterotoxin receptor (GUCY2C) (MFI score of 84%), the complement component 1q subcomponent-like 4 (C1QL4) (MFI score of 76%), the matrix remodeling associated protein 8 (MXRA8) (MFI score of 66%), the complement component 1q subcomponent B (C1QB) (MFI score of 61%) and the bone sialoprotein 2 (IBSP) (MFI score of 59%). Interestingly FLIPr showed signals of medium intensity also with the complement component 1q subcomponent A (C1QA - MFI 24%), the complement component 1q subcomponent-like 2 (C1QL2 - MFI 16%) and the complement component 1q subcomponent C (C1QC – MFI 13%). Most identified interactions are novel and require further validation since they involved loosely characterized proteins. Since the interaction between FLIPr and the human C1q subcomponents involved key players of the bacterial offense and host defense, this interaction was further validated at the biochemical and functional level.

### FLIPr binds *in vitro* the C1q subcomponents and the C1q complex purified from human plasma

**Figure 3:**
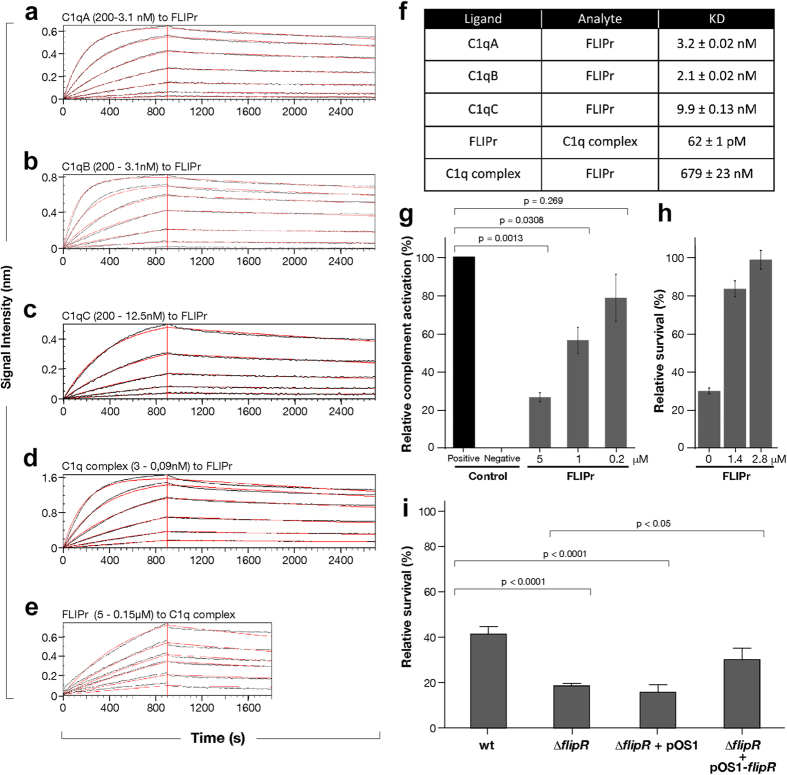
Biophysical and functional characterization of the FLIPr-C1q interaction. Bio-layer interferometry (BLI) blank subtracted sensograms of **(a)** C1qA (200-3.1 nM), **(b)** C1qB (200-3.1 nM) and **(c)** C1qC (200-12.5 nM) subcomponents tested on covalently immobilized FLIPr. **(d)** BLI blank subtracted sensograms of FLIPr (5-0.15 μM) on immobilized C1q complex and **(e)** of C1q complex (3-0.09 nM) on immobilized FLIPr. The amount of ligand associating with the analyte was measured in nanometres (nm). Association and dissociation curves were fitted in a 1:1 model. **(f)** Summary table of the measured affinity constants (KD). **(g)** Complement classical pathway influence by FLIPr in WiELISA assay. Relative % of complement activation is shown for each sample. Results are mean of three replicates. P values ≤ 0.05 were considered significant. **(h)** FLIPr-mediated dose dependent increase of *S. aureus* survival in whole blood assay. Relative survival was monitored through CFU count. **(i)** Survival in human blood of *S. aureus* USA 300 LAC wt, *flipR* deletion mutant (Δ*flipR*) and *flipR* deletion mutant complemented with empty (Δ*flipR* + pOS1) and with plasmid bearing the *flipR* insert (Δ*flipR* + pOS1-*flipR*). Results are mean of three replicates. P values ≤ 0.05 were considered significant.

To validate the FLIPr/C1q interaction the BLI (BioLayer Interferometry) technology was used. FLIPr protein was immobilized on the BLI biosensor and C1qA, C1qB and C1qC subunits, previously used in the screening, were tested as analytes in serial dilutions. The three subcomponents showed low nanomolar affinity for FLIPr (equilibrium dissociation constant, K_D_, of 3.2 nM, 2.1 nM and 9.9 nM respectively–Fig. 3a–c). In biological conditions, however, the three C1qA, C1qB and C1qC subunits are super-organized to constitute the C1q complex. Binding experiments between FLIPr and the human C1q complex purified from human plasma were therefore performed. When immobilized, FLIPr showed picomolar affinity for the C1q complex (K_D_ = 62 pM–Fig. 3d). Conversely, when the C1q complex was immobilized, the measured affinity was in the high nanomolar range (K_D_ = 679 nM–Fig. 3e).

### FLIPr reduces classical complement pathway activation and increases *S. aureus* survival in whole human blood (WHB)

In order to clarify whether the binding of FLIPr to C1q could influence activation of the complement classical pathway, a WiELISA (Wieslab) complement system screening kit was used. The complement classical pathway was investigated, and different amounts of FLIPr were pre-incubated with human serum. FLIPr at 1 μM reduced the complement classical pathway activation to ~56% and to ~26% when used at 5 μM (Fig. 3 g). FLIPr expression in *S. aureus* bacterial supernatant was confirmed by western blot assay using anti-FLIPr antibodies (Supplementary Fig. S2). To improve the understanding in FLIPr contribution to bacterial pathogenesis, *S. aureus* survival in whole human blood (WHB) was monitored in presence and absence of FLIPr. WHB pre-incubation with FLIPr significantly increased *S. aureus* survival (Fig. 3 h): FLIPr at 1.4 μM and 2.8 μM lead to a *S. aureus* survival rate of respectively 80% and 100%. A *S. aureus* knock out (KO) mutant for FLIPr was generated in order to corroborate the survival assay in whole blood. Loss of FLIPr resulted in a significant reduction of bacterial viability in WHB relative to the wild-type strains. Conversely, introduction of a plasmid borne copy of *flipR* restored viability of the bacteria compared to either the Δ*flipR* mutant or the Δ*flipR* mutant harboring the empty control plasmid (Fig. 3i).

### Screening of NadA by protein microarray revealed 8 novel putative human interactors

**Figure 4:**
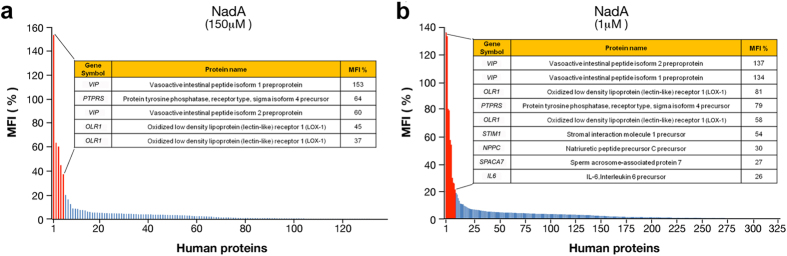
Screening for NadA interactors. **(a)** Plotting of MFIs relative percentage of spotted human proteins tested against NadA at 150 nM and **(b)** 1 μM. Red bars represent human proteins above the 25% cut-off threshold defining relevant interactions. Blue bars represent human proteins above the 4% cut-off threshold. Inset table shows gene symbol, protein name and MFI values of the relevant interactions.

In order to identify human proteins interacting with the Neisserial adhesin NadA^12^, a large-scale protein microarray screening was performed spotting 2354 human recombinant proteins from the GNF library. Biotinylated NadA was used at 150 nM and 1 μM, and interactions were detected using fluorescently labeled streptavidin. The obtained MFI was normalized for each of the spotted proteins and expressed as a percentage of the reference spots (biotinylated BSA). The results of the screening at 150 nM showed several putative human interactors (Fig. 4a). In particular, isoform 1 and 2 of the Vasoactive Intestinal Peptides (VIP) showed an MFI score of 153% and 60% respectively, and the Protein Tyrosine Phosphatase Receptor type Sigma isoform 4 (PTPRS) with an MFI of 64% were highlighted as strong hits. Two replicates of the low-density oxidized lipoprotein receptor 1 (LOX-1) showed an MFI score of 45% and 37% represented potentially relevant hits. These data were substantiated when NadA was tested at 1 μM. All five hits identified in the 150 nM screen were confirmed (Fig. 4b). In addition to these proteins, further putative hits were identified: the Stromal interaction molecule 1 precursor (STIM1), the Natriuretic Peptide Precursor C (NPPC), the sperm acrosome-associated protein 7 precursor (SPACA7) and the Interleukin 6 precursor (IL-6). Among all the identified hits, LOX-1 represented an interesting candidate for further validation processes since it is known to be involved in bacterial adhesion and invasion^20^,^21^ and its deletion enhances bacterial clearance^22^.

### LOX-1 binds to the N-terminal region of NadA

**Figure 5:**
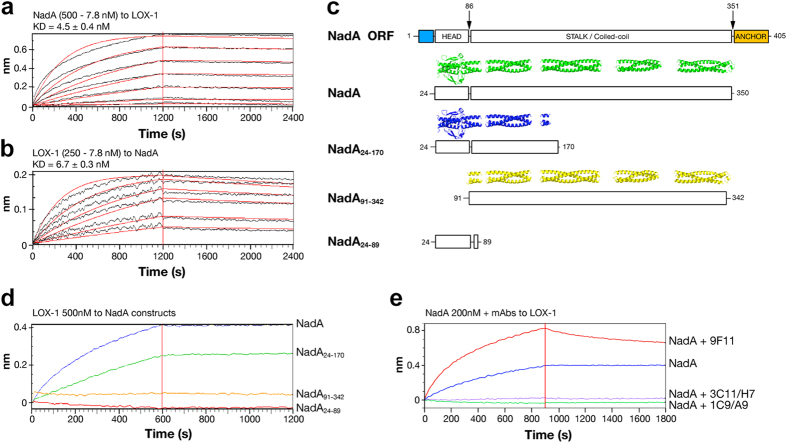
Biophysical characterization of the NadA/LOX-1 interaction. (**a)** Blank subtracted sensograms of NadA (500 - 7.8 nM) tested on covalently immobilized LOX-1 and **(b)** of LOX-1 (250 - 7.8 nM) tested on biotinylated NadA immobilized on SA biosensors. Association and dissociation curves were fitted in a 1:1 model. **(c)** Schematic representation of the NadA constructs used to determine NadA binding site on LOX-1. NadA gene, NadA (green), NadA_24–170_ (blue) and NadA_91–342_ (yellow) structure are shown. NadA_24–89_ head construct is represented only as a cartoon (rectangles) since it is not trimeric and not folded. Signal peptide (light blue) and membrane anchor (orange) are shown in the cartoon. **(d)** Blank subtracted sensograms of LOX-1 (200 nM) on biotinylated NadA (blue), NadA_24–170_ (green), NadA_91–342_ (yellow) and NadA_24–89_ (red). **(e)** Binding competition between LOX-1 and anti-NadA mAbs. Binding between NadA and LOX-1 is shown (blue). mAb 9F11 mapping NadA stalk does not inhibit the binding with NadA (red). mAb 3C11/H7 (violet) and mAb 1C9/A9 (green) mapping on NadA head abrogated NadA binding on LOX-1.

To validate and characterize the binding kinetics between NadA and LOX-1 BLI was used. LOX-1 recombinant protein was first immobilized onto the BLI biosensor, and NadA was used as analyte. The calculated K_D_ was approximately 5 nM (Fig. 5a). This value was also confirmed when the system was reverted (Fig. 5b). Dynamic light scattering (DLS) analysis was carried out to study the interaction in solution (Supplementary Fig. S3): the proteins were first analyzed alone, resulting in a hydrodynamic radius of 4.2 nm and 6.2 nm for LOX-1 and NadA, respectively. The two proteins were then mixed at a final concentration of 5 μM (each component) in a 1:1 molar ratio (trimeric NadA and dimeric LOX-1) prior to DLS analyses. As a result, a single peak with hydrodynamic radius of 8.9 nm and low polydispersity index, *i.e.* 13.4% was obtained. Nevertheless, the rod-shaped structure of both NadA and LOX-1 may have led to an overestimation of the hydrodynamic radii and therefore also to an overestimation of the protein MWs, thus hindering a precise determination of the size and stoichiometry of the complex. However, the low polydispersity and the absence of peaks corresponding to single protein radii or aggregates in the complex solution, is consistent with a 1:1 complex between one NadA trimer and one LOX-1 dimer.

To identify the binding region for LOX-1, the NadA_24–170_ (head&neck), NadA_91–342_ (stalk) and NadA_24–89_ (head-only) constructs, designed based on the recently published crystal structure^16^, were produced as his-tagged recombinant proteins (Fig. 5c). The NadA_24–170_ and NadA_91–342_ constructs were shown to be trimeric and well-folded according to size-exclusion high-performance liquid chromatography (SE-HPLC) with Multi-angle laser light scattering (MALLS), while the NadA_24–89_ resulted monomeric and unfolded (Supplementary Fig. S4), also in accordance with differential scanning calorimetry (DSC) experiments reported previously^16^. BLI binding experiments revealed the retained capability of the NadA_24–170_ construct to bind LOX-1. The binding was instead not observed for the NadA_91–342_ and NadA_24–89_ constructs (Fig. 5d). Binding competition experiments using BLI were also performed in order to understand the potential ability of anti-head monoclonal antibodies (mAbs) to inhibit the NadA/LOX-1 interaction. Anti-NadA mAbs were selected based on their binding site on NadA constructs using dot blot (Supplementary Fig. S5). BLI binding assay (Fig. 5e) showed a marked ability of mAbs (3C11/H7 and 1C9/A9) mapping to the NadA head&neck region to inhibit the binding with LOX-1. By contrast the 9F11 mAb, which maps to the NadA stalk, did not inhibit the binding to LOX-1, confirming the NadA head&neck region as the one involved in LOX-1 binding.

### NadA binds LOX-1 transfected CHO cells

**Figure 6:**
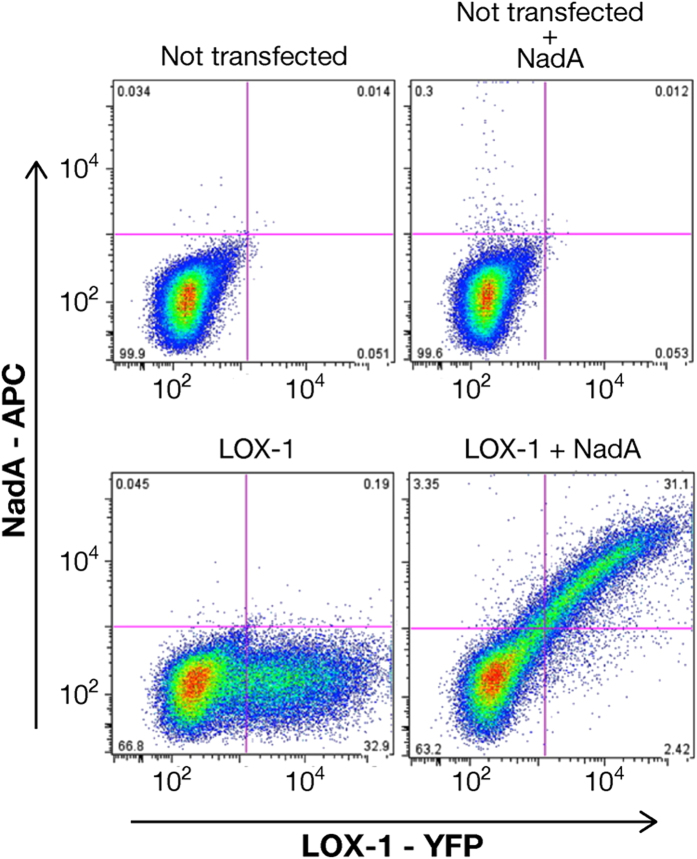
NadA binds CHO cells expressing hLOX-1. FACS plots representative of LOX-1 (YFP) expression in CHO cells and cells able to bind NadA (APC). The gating strategy was designed to separate the cells of interest from large aggregates and debris [initial gate on forward scatter (FSC) versus side scatter (SSC) plot], deplete dead cells (Live/Dead Aqua staining) and doublets/aggregates (standard gates on both FSC-width and SSC-width) (data not shown). Simple gating by quadrants allowed defining the absolute percentages of cells positive for LOX-1 only (32%, bottom left) and double positive cells (30%, bottom right) representing the cells able to bind recombinant NadA. The upper right panel shows that not-transfected cells are not able to bind NadA. The upper left panel shows the non-transfected cells.

To further validate the interaction between LOX-1 and NadA, binding assays on eukaryotic cell lines were performed. Western blot analysis confirmed that CHO (Chinese Hamster Ovary) cells do not constitutively express LOX-1 (data not shown). CHO cells were transiently transfected with a pEYFP-N1 vector expressing human LOX-1 and FACS analysis showed that transfected cells exposed the receptor on the cell surface (Fig. 6, bottom left panel). Non-transfected cells did not bind NadA (Fig. 6, upper right panel). NadA binding was observed selectively on the entire LOX-1 expressing cell population (Fig. 6, bottom right panel). These data are consistent with a specific interaction of NadA with human LOX-1 recapitulating in the cellular context the results obtained with protein array and BLI analysis.

## Discussion

*S. aureus* and *N. meningitidis* possess a wide variety of molecules specifically produced to adhere to and penetrate host tissues, evade the immune system and invade the host during pathogenesis. The main players in these processes are the host and bacterial extracellular proteomes. Recent studies pointed out the importance of a deeper understanding of the molecular mechanisms which underlie bacterial immune evasion and pathogenesis^23^. To this aim, we have applied a systematic large-scale protein microarray-based approach to identify novel host-pathogen interactions.

*S. aureus* is considered a master of complement evasion, with an impressive ability to persist within diverse microenvironments in the human body by using a number of secreted proteins that contribute to its pathogenesis. In order to discover novel interactions between the human and the *S. aureus* extracellular proteins, we screened 159 extracellular *S. aureus* proteins against 75 human extracellular components consisting of key effectors of the innate immune system as well as extracellular matrix proteins. The screening resulted in the identification of a novel target for a well-known *S. aureus* effector. We report for the first time the identification and biochemical/functional characterization of the interaction between FLIPr, a protein already described to be involved in *S. aureus* immune evasion process^24^, and the subcomponents of human complement factor C1q.

When tested by label-free BLI technology, C1qA, C1qB and C1qC showed low nanomolar equilibrium dissociation constants with FLIPr, indicating a high affinity interaction with each of the subcomponents. Additional binding experiments were performed using the assembled C1q complex purified from human plasma, confirming that FLIPr is able to bind the assembled complex, and not only its single recombinant subcomponents. The measured affinity for the interaction between FLIPr and C1q complex was different depending on the binding orientation. The reason for the latter discrepancy was not investigated. It is conceivable that since the C1q complex likely possesses multiple binding sites for FLIPr, the apparently tighter binding when FLIPr is immobilized may be due to an avidity effect of the C1q complex, as previously shown for other bacterial C1q-binding proteins^25^. FLIPr is a staphylococcal virulence factor able to interfere with opsonophagocytosis by targeting the Fcγ receptor (FcγR) and formyl-peptide receptor (FPR) on phagocytes^24^,^26^. Our data show the human complement C1q complex as an additional target for this important protein; we show that FLIPr is able to interfere with the classical complement pathway activation in a cell-free complement assay in presence of human serum and to increase *S. aureus* survival in blood, while loss of FLIPr resulted in a significant reduction of bacterial viability in WHB relative to the wild-type strains. This latter result may, in the first instance, be considered the result of a synergistic effect between the simultaneous inhibition of FcγR and FPR on phagocytes surface, and the inhibition of the complement mediated killing through C1q binding. Despite this, the phagocytosis inhibition exerted by FLIPr has been shown to dramatically decrease at near-physiological serum concentrations^24^. This effect might be described with a complement receptor-dependent phagocytosis, while only at low serum concentrations the process would be dependent on FcγR. Since the conditions we used presuppose physiological serum concentrations, we can exclude that the increased survival we see is dependent on decreased phagocytosis (dependent on FPR and Fcγ receptor). Rather, we think that the increased survival depends on complement perturbation and therefore on the interaction between FLIPr and C1q. What we hypothesize, combining the literature data and the data shown in this work, is a dual role of FLIPr during infection and pathogenesis; on one hand, in those environments at low serum concentrations like the interstitial tissue, through the binding of FcγR and FPR, FLIPr would allow to evade phagocytosis. On the other hand, in serum rich environments like the bloodstream, through the binding of C1q, FLIPr would instead allow to escape complement and complement receptor-mediated killing, but not phagocytosis. Moreover, an intracellular lifestyle at this last step may constitute a preferential mechanism for *S. aureus* survival and spreading.

As a further proof-of-concept of the experimental approach, a completely unbiased large-scale screening was carried out in order to identify novel human receptors for the Neisserial adhesin NadA by screening a library of 2354 human extracellular proteins spotted on slides. NadA is considered to be involved in the initial adhesion to epithelial cells^13^, but may act also on different cell types, including monocytes/macrophages^27^. It has also been recently reported to bind human β1 integrins that are widely distributed in different human cells^15^. Interestingly, the microarray screening here described revealed new putative NadA receptors involved in endothelial permeabilisation and bacterial pathogenesis, *i.e.* the VIP peptides and the inducible oxidized LDL receptor LOX-1^21^,^22^,^23^. LOX-1, in particular, is known to be involved in the translocation of low-density lipoprotein vesicles and even bacteria across the endothelial barrier^20^.

BLI binding experiments carried out using NadA stalk and head&neck constructs identified the latter as the major binding region. This data was also confirmed by competition experiments using anti-NadA mAbs. The mAbs targeting NadA head showed competition with LOX-1 for NadA binding. Furthermore, the binding test with the unfolded NadA_24–89_ construct revealed that a structured head domain is required to accomplish the binding, suggesting a conformation-dependent protein-protein interaction. The binding between LOX-1 and NadA was further corroborated by cellular binding assay in which the CHO cells transiently expressing LOX-1 were able to bind recombinant NadA.

The identification of the interaction between NadA and LOX-1 was reported here for the first time, and suggests an intriguing new role for the Bexsero^®^ vaccine component NadA in meningococcus B pathogenesis. In fact, NadA has been described so far as having mainly epithelial targets, with a role in the colonization/invasion of the epithelial barrier. Here we report the identification of its first endothelial receptor, LOX-1, mainly reported as the receptor for oxidized low-lipoproteins and responsible for their internalization in endothelial cells. Recently, a specific interaction between the meningococcal pili type IV and host CD147 has been reported to be essential for meningococcal adhesion to human endothelial cells and colonization of human blood vessels^28^, but other receptors might be necessary to allow bacterial invasion and pathogenesis. Meningococcus B might take advantage of the internalization mechanism exerted by LOX-1 to cross the blood-brain barrier, as also previously described for other pathogens^20^,^21^,^29^. Although further studies are necessary for a deeper understanding on NadA/LOX-1 role in endothelial crossing, our findings indicate that NadA is actually active far beyond the first meningococcal pathogenesis step and can have a crucial role at a later stage in the blood stream.

To our knowledge, this is the first time a microarray approach has been applied to the investigation of staphylococcal and meningococcal proteins targeting the human extracellular proteome. Moreover, this is the most extensive study carried out so far on protein-protein interactions at the host-pathogen interface for any human pathogen, including bacteria and parasites, raising the number of human proteins screened from a few tens up to more than 2300 that has driven the identification of novel host-pathogen interactions, providing novel insights on the molecular events that underlie *S. aureus* and *N. meningitidis* pathogenesis.

## Methods

### Selection, Cloning and Purification of SA Surface Proteins

The staphylococcal proteins printed on the arrays belong to the *S. aureus* strain NCTC 8325 and were selected *in silico* using a combined bioinformatics approach. They were first selected for the presence of extracellular signals, *e.g.* signal peptides, lipoprotein motifs, transmembrane regions and cell wall anchoring sequences–the so-called “surfome” and “secretome”^17^–and then for the presence of “protectome” signatures, *i.e.* specific functional/structural features occurring in bacterial vaccine protective antigens^18^.

Genomic DNA coding for the mature portion of the *S. aureus* proteins were cloned into the pET21b expression vector (Novagen) after PCR amplification using *Escherichia coli* BL21(DE3) (Novagen) as competent strain. Recombinant proteins (108 as single constructs and 10 as multiple constructs for a total of 159), obtained as C-terminal His or Glutathione *S*-transferase (GST) tag fusions, were expressed in High Throughput Medium Complex (HTMC) (3% yeast extract, 40 mM KH_2_PO_4_, 90 mM K_2_HPO_4_, 2 mM MgSO_4_, 1.5% glycerol, pH7.4) auto-inducing medium for 30 h at 27 °C and cell were then harvested at 6500 × g for 1 h. Bacterial lysis was obtained using B-PER buffer (Thermo Scientific) and the lysate was clarified by centrifugation at 30.000 × g for 30 min. The soluble fraction was loaded onto a His Multitrap HP 96-well plate system (GE Healthcare), washed with phosphate buffer (50 mM sodium phosphate, 300 mM NaCl, 20 mM imidazole pH 8.0) and the protein was finally eluted with the same buffer containing 250 mM imidazole as previously described^30^. GST tagged proteins were purified using a GST Multitrap HP 96-well plate system (GE Healthcare), washed with phosphate buffer (50 mM Tris, 300 mM NaCl pH 8.0). The proteins were eluted with the same buffer containing 10 mM reduced glutathione. Proteins purified in a tagless form were obtained as described by Klock *et al*. (27). Briefly, the PCR product of the portion of the gene coding for the mature protein was cloned using the Polymerase Incomplete Primer Extension (PIPE) method into plasmid pSpeedET, which encodes an expression and purification tag followed by a tobacco etch virus protease site (MGSDKIHHHHHHENLYFQG) at the N terminus of the protein. *E. coli* strain HK100 was transformed with the plasmid coding for the protein and was grown in Luria-Bertani (LB) arabinose-containing broth until OD_600_ = 0,4–0,6. Protein expression was achieved using IPTG (Isopropyl-β-D-thiogalactopyranosid) 1 mM for 3 h at 25 °C. Cells were harvested at 6500 × g for 1 h and lysed in B-PER buffer (Thermo Scientific). Lysate was clarified by centrifugation at 30.000 × g for 30 min and the protein contained in the soluble fraction was purified using an automated AKTA x-PRESS system on a nickel affinity column, followed by a desalting step using 3 × 5 ml HiTrap Desalting columns equilibrated in 50 mM HEPES pH 8.0, 1 mm tris(2-carboxyethyl)phosphine (TCEP). Purified protein was digested overnight at 4 °C with 1 mg of tobacco etch virus (TEV) protease/10 mg of eluted protein and was passed over a nickel affinity column collecting the flow-through. Purified proteins were stored at −20 °C and analysed by SDS-PAGE and Matrix-Assisted Laser Desorption Ionization-Time Of Flight (MALDI-TOF) to assess their integrity, identity, and purity level (range of purity 60–90%).

### Staphylococcal Surface Protein Microarray construction, imaging and data analysis

The Staphylococcal protein microarray was generated by spotting purified recombinant proteins (0.5 mg/ml in a final glycerol concentration of 40%) in 16 replicates on nitrocellulose coated slides (FAST slides; Whatman) using the ink-jet spotter Marathon (Arrayjet) resulting in spots of ~110 μm in diameter. Printing was performed in a cabinet with controlled humidity and temperature (55–60% and 12 °C respectively). A standard curve made of eight concentrations (twofold dilution from 0.5 to 0.004 mg/ml) of an amino terminal FLAG-tagged protein (P7582 Sigma) was used to assess comparison of replicate experiments (Supplementary Fig. S6A). Mean fluorescence intensities (MFI) of FLAG-tagged protein spots obtained after detection with Cy5-conjugated anti-FLAG were fitted best by sigmoid curves, showing a signal dynamic range of about 2 logs of fluorescence intensity values and a lower detection limit corresponding to *ca.* 0.03 ng (Supplementary Fig. S6B). The MFI value (63000) of the two highest points of the control dots was arbitrary set as 100%. For each of the spotted protein, the obtained MFI was normalized and expressed as a percentage of the reference spots using the following formula: (MFI of the protein × 100)/63000. Fluorescent BSA Cy3/Cy5 conjugated at 0,5 mg/ml and a standard curve of mouse IgGs were used to obtain eight replicates of a fluorescence standard curve to assess array coordinates. Meningococcal factor H binding protein (fHbp) was printed as a positive control for interaction with human factor H.

Each *S. aureus* protein was printed in 16 replicates all over the slide in a random fashion to minimize detection of false-positives that might occur by possible contamination between adjacent spots. Preliminary slide validation experiments, in which the slides were probed with mouse anti-GST and anti-6xHis monoclonal antibodies followed by detection with a Cy5-conjugated anti-mouse IgG secondary antibody, were performed to confirm the efficiency and reproducibility of the protein deposition on the chips.

Nonspecific binding was minimized by preincubating arrays with a blocking solution containing NAP blocker (G-Bioscences) diluted 1:3 in PBS (Nap-PBS) for 1 hour. Human tagged proteins were diluted in Nap-PBS at final concentration of 10 μg/ml and overlaid on the arrays (1 μg protein per slide) at RT for 1 h. After washing with 0.1% Tween 20 in PBS buffer (TPBS), arrays were incubated with mouse anti-FLAG (1:200– Sigma Aldrich) at RT for 1 h. Slides were washed again as before and interactions were detected by incubating with a Cy5 labeled anti-mouse antibody (Jackson Immunoresearch). Fluorescence images were obtained using Power scanner (Tecan Trading AG, Switzerland) and the 16-bit images were generated with PowerScanner software v1.2 at 10 μm/pixel resolution and spot fluorescence intensities were determined using ImaGene 7.0 software (Biodiscovery Inc.). Microarray data analysis was performed using in-house developed software. For each protein, the mean fluorescence intensity (MFI) of replicated spots was determined, after subtraction of the background value surrounding each spot.

MFI values greater than 3000 (equal to the mean signal of the controls spots after detection with anti-Flag and anti-mouse alone, plus ten times standard deviation values) were considered as a positive interaction. Three arbitrary MFI thresholds were also assigned for low (3000 to 15000 MFI), medium (15000 to 30000 MFI) and high (30000 to saturation) reactivity between two interacting proteins.

Negative control experiments were represented by slides incubated with anti-FLAG and anti-mouse antibodies only; the measured signals for each spot of the negative control slide was subtracted from those obtained for the same spot in the slides probed with human proteins. The average of the 16 spots replicates constitutes the mean fluorescent intensity (MFI) value taken in consideration for the validation experiments.

### FLIPr expression and purification

To obtain an amount of protein in a mg range, FLIPr (25–133) was expressed in BL21 E. *coli* (DE3) cells in HTMC autoinducing media (3% yeast extract, 40 mM KH_2_PO_4_, 90 mM K_2_HPO_4_, 2 mM MgSO_4_, 1.5% glycerol, pH7.4). Bacteria were grown for 30 h at 27 °C and cell were then harvested at 10000 × g for 1 h. Bacterial pellets were suspended in lysis buffer (50 mM NaH_2_PO_4_ pH 8,2, 300 mM NaCl supplemented with Roche EDTA-free protease inhibitors) lysate was clarified by centrifugation at 30.000 × g for 30 min. The histidine-tagged protein was purified using a nickel column (His Trap FF, 5 ml–GE Healthcare) following the manufacturer’s instructions. An additional step on Superdex 75 26/60 (GE Healthcare) was performed to remove possible aggregates. Sample purity was checked by 4–12% SDS-PAGE and SE-UPLC.

### Construction of a *flipR* mutant

Primers for mutant and complementing plasmid construction are listed in Table 1. For the construction of an unmarked in-frame deletion mutant in *flipR* (NWMN_1067), a 1004 bp long fragment upstream of *flipR* and comprising the first six nucleotides of the gene as well as a 1029 bp fragment downstream of *flipR* and comprising the last six nucleotides of the gene was amplified from *S. aureus* strain Newman by PCR using KAPA HiFi DNA polymerase (Kapa Biosystems is based in Wilmington, Massachusetts, USA) and primers NWMN_1067_−998_KpnI_F2 and NWMN_1067_ +6_R or primers NWMN_1067_ +397_F and NWMN_1067_ +1425_SacI_R2, respectively. The two flanking regions were then fused by PCR using primers NWMN_1067_−998_KpnI_F2 and NWMN_1067_ +1425_SacI_R2 by combining equimolar ratios of the flanking regions and cycling without primers for five cycles followed by another 30 cycles after addition of the primers. The resulting product was digested by *Kpn*I and *Sac*I, cloned into pIMAY^31^ passaged through RN4220 and transformed into the *S. aureus* USA300 strain LAC. Recombination and excision of the plasmid were performed as described previously^31^. In brief, the transformed strains were grown in TSB in the presence of 10 μg ml^−1^ chloramphenicol at 28 °C and 250 rpm overnight and integration of the plasmid into the genome triggered by incubating overnight at 37 °C and 250 rpm. The culture was then streaked out onto TSA + chloramphenicol and incubated overnight at 37 °C. To promote excision of the plasmid cultures were then grown at 28 °C for 8 h and serial dilutions were plated onto TSA supplemented with 1 μg ml^−1^ anhydrotetracycline to select for loss of the plasmid. Ten large single colonies were screened by colony PCR using primers NWMN_1067_−1144_F and NWMN_1067_ + 1542_R confirming the deletion of *flipR*. Loss of the excised plasmid was confirmed by plating onto TSA alone and TSA supplemented with chloramphenicol.

### Complementation of the *flipR* mutant

In order to complement the *flipR* deletion mutant, the *flipR* gene including 313 bp upstream of the start codon was amplified using primers NWMN_1067_−313_EcoRI_F and NWMN_1067_ + 467_PstI_R (Table 1) and cloned into the *EcoR*I and *Pst*I restriction sites of pOS1^32^. The complementing plasmid and the empty control plasmid were passaged through RN4220 and then transformed into *S. aureus* strain USA300 Δ*flipR*.

### Assessment of *in vitro* complement activity

The assessment of *in vitro* complement activity was analysed using a complement screening kit (Wieslab COMP300). This enzyme based immunoassay was developed for the *in vitro* determination of complement dysfunctions in patients. The system relies on the specific activation of the three distinct complement pathways (CP, MBL pathway and AP) through activators immobilized on the microtiter wells, using the deposition of the terminal MAC (C5-9) complex as readout. Buffers containing specific blockers to ensure the activation of the respective pathway were used to dilute the samples. For our purposes, serial concentrations (5 µM, 1 µM and 0.2 µM) of FLIPr in HBS buffer (20 mM HEPES, 150 mM NaCl pH 7.3) were added to human serum previously diluted as suggested by supplier. Positive control was constituted by diluted serum and HBS buffer only, while negative control was constituted by heat inactivated serum. Additional control is represented by HBS buffer pre-incubated with serum. The amount of complement activation correlates with the colour intensity measured as OD_405_ nm. The value for the positive control was defined as 100%; the value for negative control as 0%. All measured values were expressed as relative % of complement activation using the following formula: (sample value − negative control value)/(positive control value−negative control value) × 100. Student’s t test was used for comparison of paired means of two groups. P values of 0.05 were considered significant.

### Whole blood phagocytosis assay

*S. aureus* survival in presence of FLIPr was assessed through whole blood phagocytosis assay. For such purpose an overnight culture of *S. aureus* cells from USA 300 LAC strain in BHI, was diluted 1:40 and grown at 37 °C with shaking 180 RPM to OD_600_ = 0.300. The bacteria were then diluted in BHI and 50 μl of the bacterial suspension (2.5 × 10^5^CFU) were added to 1 ml of fresh blood from healthy donors supplemented with anticoagulant lepirudin 50 mg/L (20 μl/ml of blood) and previously preincubated for 30 min at 37 °C with different amount of FLIPr (0.4-3 μM), or with FLIPr KO (Δ*flipR*), or the Δ*flipR* mutant harboring the empty control plasmid or with PBS. A portion of this culture was plated on BHI-agar to determine the input CFU. The blood samples were incubated with bacteria at 37 °C for 2 h with shaking 180 RPM. Aliquots were then incubated for 3 min on ice with a final concentration of 0.5% of saponin-PBS to lyse neutrophils. The number of viable bacteria was determined by serial tenfold dilutions in BHI and plating on BHI- agar plates. Colonies were counted after incubation of the plates at 37 °C for 18 h. Control was the blood sample preincubated with PBS. Normal freshly drawn human blood was obtained from healthy volunteers and informed consent was obtained from all participants. The methods described were carried out in accordance with the approved guidelines and experimental protocols were approved with the recommendations of the local ethical committee of the University of Pavia (permit 19/9/2013).

### Bacterial supernatant analysis

The determination of FLIPr in bacterial supernatants has been evaluated by western blot analyses. *Staphylococcus aureus* Newman WT was grown overnight in TSB (17 g/l bacto tryptone, 3 g/l bacto soyone, 2.5 g/l glucose, 5 g/l NaCl, 2.5 g/l Dipotassium Hydrogen Phosphate) medium at 37 °C until it reached the OD_600_ = 11,7 (stationary phase) and cell-free supernatant was harvested by centrifugation and filter sterilized. For the western blot analyses, supernatant has been concentrated 40 times with Amicon CutOff 3 kDa and protein content quantify by BCA assay (Thermo Scientific). 5 μl of concentrated supernatant have been loaded into a 4–12% SDS-PAGE gel (Invitrogen) and western blot assay was performed using affinity purified monoclonal antibody against FLIPr produced in mouse and anti-mouse HRP 1:5000.

### GNF Protein microarray construction and processing

The GNF protein microarrays were generated by using 2354 human recombinant proteins from the GNF library, which were produced as described previously^7^. Each protein of the library was spotted in duplicates in 24 blocks per array onto ultra-thin nitrocellulose coated glass slides (PATH slides; Grace Bio Labs) using a contact printer (MicroGrid II; Digilab). Printing was performed at room temperature and ambient relative humidity. Proteins were diluted ½ in arraying buffer (100 mM NaCl, 0.05% TritonX and 5% Glycerol in PBS) and printed between 10 amol to 75 fmol per 150 μm diameter spot. A Chrompure human IgG (Jackson IR 009-000-003) was printed at 0.4 fmol per spot several times on each single block and served as a printing control and to define the blocks. Eight empty blocks were available to print additional antigens and relevant controls such as biotinylated BSA. Since all secretomics proteins have a 6-His tag and approximately 1/3 have an additional mouse Fc-tag, the printing efficiency of the proteins and microarray quality was evaluated by probing the microarrays with a mouse anti-His (Sigma H1029) antibody followed by a detection using a mix of a Cy5 conjugated F(ab′)_2_ goat a-mIgG Fc specific (Jackson IR 115-176-071) and a Cy5 conjugated goat α-hIgG F(ab′)_2_ specific secondary antibody (Jackson IR 109-175-097). The last secondary antibody (Jackson IR 109-175-097) was systematically used in the assays to detect the human IgG control spots printed on the arrays in order to define the blocks. Bexsero^®^ vaccine antigen NadA, was biotinylated using EZ-Link Sulfo-NHS-LC-Biotin (Thermo Scientific) following manufacturer’s instruction. The mass of the proteins and degree of biotinylation was checked with Liquid chromatography coupled to mass spectrometry (LC-MS). The incubation of protein microarrays with NadA was performed using an automated hybridization station (HS400; Tecan), programmed with an internal protocol. First, the protein microarrays were incubated with a PBS blocking solution containing 1% BSA and 0.1% Tween20 for 1 hour at 23 °C to reduce non-specific binding. Then, NadA, diluted in probing buffer (2.5% glycerol, 1% BSA, and 0.05% TritonX in PBS) at final concentrations of 150 nM and 1 μM, were incubated for 1 h at 23 °C. After a washing step with probing buffer, the microarrays were probed with Cy5-Streptavidin (Invitrogen SA1011) combined with Cy5-goat a-hIgG F(ab′)_2_ specific (Jackson IR 109-175-097) diluted in probing buffer at 7.5 μg/ml final concentration. Finally, after two washing steps using probing buffer and Milli-Q water, the microarrays were dried under a flow of nitrogen at 30 °C for 4 min. In parallel a control microarray slide probed with buffer and the mix of the detection antibodies was performed.

### GNF Microarray imaging and data analysis

Microarray image acquisition was done using a fluorescence microarray scanner (GenePix Professional 4200 A; Molecular Devices) equipped with a red laser (635 nm) at a resolution of 10 μm/pixel and images were analyzed using the GenePix Pro v6.1 software (Molecular Devices). Data analysis was performed using in-house developed software. For each protein, the mean fluorescence intensity (MFI) of replicated spots was determined, after subtraction of the background value surrounding each spot. The MFI value of the control dots (biotinylated BSA) was arbitrary set as 100%. For each of the spotted protein, the obtained MFI was normalized and expressed as a percentage of the reference spots. Proteins with a high coefficient variation (CV) between the two replicates were discarded. All signals with MFI score equal or greater than 25% were considered as positive hits, excluding the ones found in the control microarray where the Cy5-Streptavidin alone was probed to determine its non-specific binding to the printed proteins.

### NadA and NadA constructs cloning, expression, purification and characterization

The *nadA* gene fragments from *N. meningitidis* strain 2996 were cloned by PCR, using the PIPE (polymerase incomplete primer extension) method, as described previously^16^. The cloned fragments lacked the first 23 residues of NadA which encode a signal peptide for protein export. The fragments were inserted into a pET-21 vector (Novagen), enabling cytoplasmic expression of the NadA proteins with a C-terminal 6-His tag. The residue numbering employed refers to the full-length NadA protein, Uniprot code Q8KH85. All NadA proteins were produced in *E. coli* BL21D3 (T1r) cells (Invitrogen) and were purified at room temperature (RT, 18–26 °C) using an AKTA purifier 10 system (GE Healthcare) by Ni-affinity chromatography (5 mL HiTrap Ni-NTA column) and by size-exclusion chromatography on a HiLoad (16/60) Superdex 75 column equilibrated in 20 mM Tris–HCl, 150 mM NaCl pH8.0. The quality of the final NadA samples was checked using 4–12% SDS–PAGE gradient gels in MES buffer and also by size-exclusion high-performance liquid chromatography (SE-HPLC), revealing a high level of purity (estimated to be >97% for all proteins) and a lack of any aggregated species. SE-HPLC was performed at RT on an analytical size exclusion TSK Super SW3000 column by loading 20 μl of each sample at a concentration of ~40 μM. Samples were eluted isocratically in 0.1 M NaH_2_PO_4_, 0.4 M (NH_4_)_2_SO_4_ buffer at pH 6.0. Coupling SE-HPLC with Multi-angle laser light scattering (SE-HPLC/MALLS) NadA samples were analyzed for molecular size. Data analyses were carried out using Astra V software (Wyatt) to determine the weight-average molecular mass (MW) in Daltons and the polydispersity index (MW/Mn) for each oligomer present in solution.

### BioLayer Interferometry (BLI)-based OCTET analysis

Octet QKe (ForteBio, Pall Science) was used for binding studies. SA (streptavidin) and AR2G (amine reactive 2nd generation) tips were obtained from ForteBio and were used respectively to immobilize biotinylated proteins and to covalently attach ligand protein using amine based chemistry. Immediately before analysis, tips were prewet in kinetic buffer.

For the FLIPr-C1q characterization, after 180 sec baseline in kinetic buffer (10 mM HEPES, 300 mM NaCl, 0,02% P-20 pH 7.4), the biosensor tips were activated for 300 sec using a freshly mixed solution of 200 mM EDC in 50 mM NHS further diluted 1:20 in dH_2_O and the tips were then immersed in ligand solution for 600 sec loading (C1q 12.5 ug/ml in 10 mM NaH_2_PO_4_ pH 6.5 or FLIPr 50 ug/ml in 10 mM Na acetate pH 5). Excess reactive groups were blocked with 1 M ethanolamine pH 8.5 for 300 sec and a second 300 sec baseline in kinetic buffer was performed and kinetic was monitored.

For the NadA/LOX-1 characterization after 180 sec baseline in kinetic buffer (10 mM HEPES, 150 mM NaCl, 0,02% P-20 pH 7), AR2G tips were activated for 300 sec using a freshly mixed solution of 200 mM EDC in 50 mM NHS further diluted 1:20 in dH_2_O. The tips were then immersed in ligand solution (LOX-1 at 12.5 μg/ml in acetate pH 5.5) for 600 sec. Excess reactive groups were blocked with 1 M ethanolamine pH 8.5 for 300 sec and a second 300 sec baseline in kinetic buffer was performed and kinetic was monitored. For NadA constructs characterization, SA biosensor tips after 180 sec baseline in kinetic buffer, were directly immersed in ligand solution for 600 sec loading (biotinylated NadA, NadA_24–170_ and NadA_91–342_ at 25 μg/ml diluted kinetic buffer) and a second 300 sec baseline in kinetic buffer was performed and kinetic was monitored. Recombinant LOX-1 ectodomain produced in mammalian cells was purchased from R&D systems.

During the entire kinetic assay, the sample plate was kept shaking 1000 rpm. A column of biosensors where no ligand protein was loaded, was activated and quenched (where necessary) to be used as parallel reference control in association with the analyte titration; a ligand-loaded biosensor was also used in association with buffer as baseline.

Reference subtracted BLI response curves were used for the affinity constant determination. Inter-step correction and Y-alignment were used to minimize tip-dependent variability. Data were globally fitted in a 1:1 model using the Data Analysis Software v7.1 (Forte Bio).

### Dot blot assays

NadA_24–170_ (head&neck), NadA_91–342_ (stalk) and NadA protein constructs (200 ng) were spotted and adsorbed onto nitrocellulose membrane strips. The strips were blocked in PBS containing 3% (w/v) milk and 0.1% Tween 20 (v/v), and then incubated with the mAbs 9F11, 1C9/A9 and 3C11/H7 at 1 μg/ml for 1 h at 37 °C, respectively. Membranes were washed with PBS containing 0.1% Tween 20 (v/v), (T-PBS), and then incubated with sheep anti-mouse horseradish peroxidase-conjugated IgG diluted 1:5000 in milk/T-PBS. Membranes were washed and signals were revealed with the SuperSignal WestPico Chemiluminescent Substrate Kit (Thermo Scientific Pierce, Waltham, MA, USA).

### mAbs selection and binding competition

For competition experiments, a preliminary screening to select high-affinity anti-NadA mAbs was performed. Protein G biosensors tips were pre-wet in kinetic buffer (10 mM HEPES, 150 mM NaCl, 0,02% P-20 pH 7.4). After 180 sec baseline, tips were directly immersed in each mAb (diluted at 50 μg/ml in kinetic buffer) for 600 sec. A second 300 sec baseline in kinetic buffer was performed. NadA was tested as analyte at 200 nM. Competition experiments were performed immobilizing LOX-1 as above. NadA was pre-incubated with an excess of mAb (1:3) overnight at 4 °C and the mix was used as analyte in the same assay conditions as above.

### Dynamic Light Scattering (DLS) measurements

For DLS analysis DynaPro^®^ Plate Reader II (WYATT Technology) with 96 Well Plates was used. NadA, LOX-1 and the complex mixed in a 1:1 molar ratio were diluted in HBS-P buffer in a final concentration of 5 μM. A cut off from 0, 1–100 nm was applied. Results are mean of 30 measurements per sample.

### FACS binding experiments

To perform binding assay, LOX-1 transfected and non-transfected CHO cells were detached using trypsin (CDS, Sigma), harvested and suspended in F12-K medium supplemented with 10% FBS. Approximately 1 × 10^6^ cells were incubated with 200 μg/ml NadA or PBS alone for 30 minutes at 4 °C. Cells were then incubated with 5 μg/ml of anti-NadA 9F11 mouse monoclonal antibody for 1 hour at 4 °C, and with Allophycocyanin (APC)-conjugated goat F(ab)2 anti-mouse Ig (diluted 1:100; Jackson ImmunoResearch Laboratories) for 30 minutes at 4 °C. Cells were stained with Live/Dead Aqua (Invitrogen) diluted 1:400 for 20 minutes in the dark and analyzed with a Canto II analyzer (Becton Dickinson, Pharmingen, San Diego, CA). Flow-cytometric analysis was performed using FlowJo software (Treestar Inc.).

## Additional Information

**How to cite this article**: Scietti, L. *et al*. Exploring host-pathogen interactions through genome wide protein microarray analysis. *Sci. Rep.* **6**, 27996; doi: 10.1038/srep27996 (2016).

## Supplementary Material

Supplementary Information

## Figures and Tables

**Table 1:** Primers used in this study.

Primer name	Sequence (5′-3′)
NWMN_1067_−998_KpnI_F2	AAGCTGGGTACCCCATTGAATTAAATGCTCTAAAACGAC
NWMN_1067_+6_R	GTATGTTTTTTAATATTTCATAATAAGTTCTCCCTGTAAAATAAATTTG
NWMN_1067_+397_F	CTTATTATGAAATATTAAAAAACATACTGAATTAAATAGTTGTACGC
NWMN_1067_ +1425_SacI_R2	GAATTGGAGCTCGATAACCCTATATGGTTGAATCATGTTG
NWMN_1067_−313_EcoRI_F	ATCCGGGAATTCGGAGTGTTTGTCTATCCAACTTAGCAAAC
NWMN_1067_+467_PstI_R	GCTTGGCTGCAGGCACGCCAATACGTTAGCATTG
NWMN_1067_−1144_F	GTGACCCAGCTAATGATTAACGTTG
NWMN_1067_+1542_R	GGAGGTCGTGTCATGAATGTTTG

## References

[b1] Bardoel B. W. & Strijp J. A. Molecular battle between host and bacterium: recognition in innate immunity. J Mol Recognit 24, 1077–1086 (2011). 22038814 10.1002/jmr.1156

[b2] Crosnier C. . Basigin is a receptor essential for erythrocyte invasion by *Plasmodium falciparum*. Nature 480, 534–537 (2011). 22080952 10.1038/nature10606 PMC3245779

[b3] Bartholdson S. J. . Semaphorin-7 A is an erythrocyte receptor for *P. falciparum* merozoite-specific TRAP homolog, MTRAP. PLoS Pathog 8, e1003031 (2012). 23166499 10.1371/journal.ppat.1003031 PMC3499583

[b4] Bartholdson S. J., Crosnier C., Bustamante L. Y., Rayner J. C. & Wright G. J. Identifying novel *Plasmodium falciparum* erythrocyte invasion receptors using systematic extracellular protein interaction screens. Cell Microbiol 15, 1304–1312 (2013). 23617720 10.1111/cmi.12151 PMC3798119

[b5] Galeotti C. L. . Surface interactome in *Streptococcus pyogenes*. Mol Cell Proteomics 11, M111 015206 (2012). 22199230 10.1074/mcp.M111.015206 PMC3322576

[b6] Margarit I. . Capturing host-pathogen interactions by protein microarrays: identification of novel streptococcal proteins binding to human fibronectin, fibrinogen, and C4BP. FASEB J 23, 3100–3112 (2009). 19417080 10.1096/fj.09-131458

[b7] Gonzalez R. . Screening the mammalian extracellular proteome for regulators of embryonic human stem cell pluripotency. Proc Natl Acad Sci USA 107, 3552–3557 (2010). 20133595 10.1073/pnas.0914019107 PMC2840467

[b8] Peacock S. J., de Silva I. & Lowy F. D. What determines nasal carriage of *Staphylococcus aureus*? Trends Microbiol 9, 605–610 (2001). 11728874 10.1016/s0966-842x(01)02254-5

[b9] Foster T. J., Geoghegan J. A., Ganesh V. K. & Hook M. Adhesion, invasion and evasion: the many functions of the surface proteins of *Staphylococcus aureus*. Nat Rev Microbiol 12, 49–62 (2014). 24336184 10.1038/nrmicro3161 PMC5708296

[b10] Kim H. K., Thammavongsa V., Schneewind O. & Missiakas D. Recurrent infections and immune evasion strategies of *Staphylococcus aureus*. Curr Opin Microbiol 15, 92–99 (2012). 22088393 10.1016/j.mib.2011.10.012 PMC3538788

[b11] Serruto D., Bottomley M. J., Ram S., Giuliani M. M. & Rappuoli R. The new multicomponent vaccine against meningococcal serogroup B, 4CMenB: immunological, functional and structural characterization of the antigens. Vaccine 30 Suppl 2, B87–97 (2012). 22607904 10.1016/j.vaccine.2012.01.033 PMC3360877

[b12] Magagnoli C. . Structural organization of NadADelta(351–405), a recombinant MenB vaccine component, by its physico-chemical characterization at drug substance level. Vaccine 27, 2156–2170 (2009). 19356620 10.1016/j.vaccine.2009.01.099

[b13] Capecchi B. . *Neisseria meningitidis* NadA is a new invasin which promotes bacterial adhesion to and penetration into human epithelial cells. Mol Microbiol 55, 687–698 (2005). 15660996 10.1111/j.1365-2958.2004.04423.x

[b14] Cecchini P. . The soluble recombinant *Neisseria meningitidis* adhesin NadA(Delta351–405) stimulates human monocytes by binding to extracellular Hsp90. PLoS One 6, e25089 (2011). 21949862 10.1371/journal.pone.0025089 PMC3175003

[b15] Nagele V. . *Neisseria meningitidis* adhesin NadA targets beta1 integrins: functional similarity to *Yersinia* invasin. J Biol Chem 286, 20536–20546 (2011). 21471204 10.1074/jbc.M110.188326 PMC3121457

[b16] Malito E. . Structure of the meningococcal vaccine antigen NadA and epitope mapping of a bactericidal antibody. Proc Natl Acad Sci USA 111, 17128–17133 (2014). 25404323 10.1073/pnas.1419686111 PMC4260552

[b17] Bagnoli F. . Vaccine composition formulated with a novel TLR7-dependent adjuvant induces high and broad protection against *Staphylococcus aureus*. Proc Natl Acad Sci USA 112, 3680–3685 (2015). 25775551 10.1073/pnas.1424924112 PMC4378396

[b18] Altindis E. . Protectome analysis: a new selective bioinformatics tool for bacterial vaccine candidate discovery. Mol Cell Proteomics 14, 418–429 (2015). 25368410 10.1074/mcp.M114.039362 PMC4350036

[b19] Kilpatrick D. C. & Chalmers J. D. Human L-ficolin (ficolin-2) and its clinical significance. J Biomed Biotechnol 2012, 138797 (2012). 22500076 10.1155/2012/138797 PMC3303570

[b20] Shimaoka T. . LOX-1 supports adhesion of Gram-positive and Gram-negative bacteria. J Immunol 166, 5108–5114 (2001). 11290792 10.4049/jimmunol.166.8.5108

[b21] Campbell L. A. . *Chlamydia pneumoniae* binds to the lectin-like oxidized LDL receptor for infection of endothelial cells. Microbes Infect 14, 43–49 (2012). 21911078 10.1016/j.micinf.2011.08.003 PMC3247659

[b22] Wu Z. . LOX-1 deletion improves neutrophil responses, enhances bacterial clearance, and reduces lung injury in a murine polymicrobial sepsis model. Infect Immun 79, 2865–2870 (2011). 21576343 10.1128/IAI.01317-10 PMC3191957

[b23] Laarman A., Milder F., van Strijp J. & Rooijakkers S. Complement inhibition by gram-positive pathogens: molecular mechanisms and therapeutic implications. J Mol Med (Berl) 88, 115–120 (2010). 20062962 10.1007/s00109-009-0572-y PMC2832872

[b24] Stemerding A. M. . *Staphylococcus aureus* formyl peptide receptor-like 1 inhibitor (FLIPr) and its homologue FLIPr-like are potent FcgammaR antagonists that inhibit IgG-mediated effector functions. J Immunol 191, 353–362 (2013). 23740955 10.4049/jimmunol.1203243

[b25] Kang M. . Collagen-binding microbial surface components recognizing adhesive matrix molecule (MSCRAMM) of Gram-positive bacteria inhibit complement activation via the classical pathway. J Biol Chem 288, 20520–20531 (2013). 23720782 10.1074/jbc.M113.454462 PMC3711317

[b26] Prat C., Bestebroer J., de Haas C. J., van Strijp J. A. & van Kessel K. P. A new staphylococcal anti-inflammatory protein that antagonizes the formyl peptide receptor-like 1. J Immunol 177, 8017–8026 (2006). 17114475 10.4049/jimmunol.177.11.8017

[b27] Franzoso S. . Human monocytes/macrophages are a target of *Neisseria meningitidis Adhesin A* (NadA). J Leukoc Biol 83, 1100–1110 (2008). 18299457 10.1189/jlb.1207810

[b28] Bernard S. C. . Pathogenic *Neisseria meningitidis* utilizes CD147 for vascular colonization. Nat Med 20, 725–731 (2014). 24880614 10.1038/nm.3563 PMC7095922

[b29] Honjo M. . Lectin-like oxidized LDL receptor-1 is a cell-adhesion molecule involved in endotoxin-induced inflammation. Proc Natl Acad Sci USA 100, 1274–1279 (2003). 12538855 10.1073/pnas.0337528100 PMC298763

[b30] Palumbo E. . Antigen identification starting from the genome: a “Reverse Vaccinology” approach applied to MenB. Methods Mol Biol 799, 361–403 (2012). 21993656 10.1007/978-1-61779-346-2_21

[b31] Monk I. R., Shah I. M., Xu M., Tan M. W. & Foster T. J. Transforming the untransformable: application of direct transformation to manipulate genetically *Staphylococcus aureus* and *Staphylococcus epidermidis*. MBio 3 (2012). 10.1128/mBio.00277-11 PMC3312211 22434850

[b32] Schneewind O., Mihaylova-Petkov D. & Model P. Cell wall sorting signals in surface proteins of gram-positive bacteria. EMBO J 12, 4803–4811 (1993). 8223489 10.1002/j.1460-2075.1993.tb06169.x PMC413927

