# Supplementary material for: Exploring host-pathogen interactions through genome wide protein microarray analysis
Source: Sci Rep. 2016 Jun 15;6:27996. doi: 10.1038/srep27996 (PMC4908583; doi:10.1038/srep27996)
Supplement: Supplementary Information [file srep27996-s1.pdf]

Exploring host-pathogen interactions through genome wide protein microarray analysis - Luigi Scietti, Katia Sampieri, Irene Pinzuti, Erika Bartolini, Barbara Benucci, Alessia Liguori, Andreas F. Haag, Paola Lo Surdo, Werner Pansegrau, Vincenzo Nardi-Dei, Laura Santini, Seguinde Arora, Xavier Leber, Simonetta Rindi, Silvana Savino, Paolo Costantino, Domenico Maione, Marcello Merola, Pietro Speziale, Matthew J. Bottomley, Fabio Bagnoli, Vega Masignani, Mariagrazia Pizza, Meike Scharenberg, Jean-Marc Schlaeppi, Mikkel Nissum and Sabrina Liberatori

Supplementary Figure S1 - **Screening results.** Classification (differentiated for each human protein class) of the MFI values based on background and thresholds. The 96% of the combinations between a *S. aureus* and human protein resulted below the background threshold (grey). The hits above 3000 MFI were classified in low (yellow - 4%), medium (orange - 0.36%) and highly reactive (red - 0.16%). Ficolin 2 was discarded from this analysis.

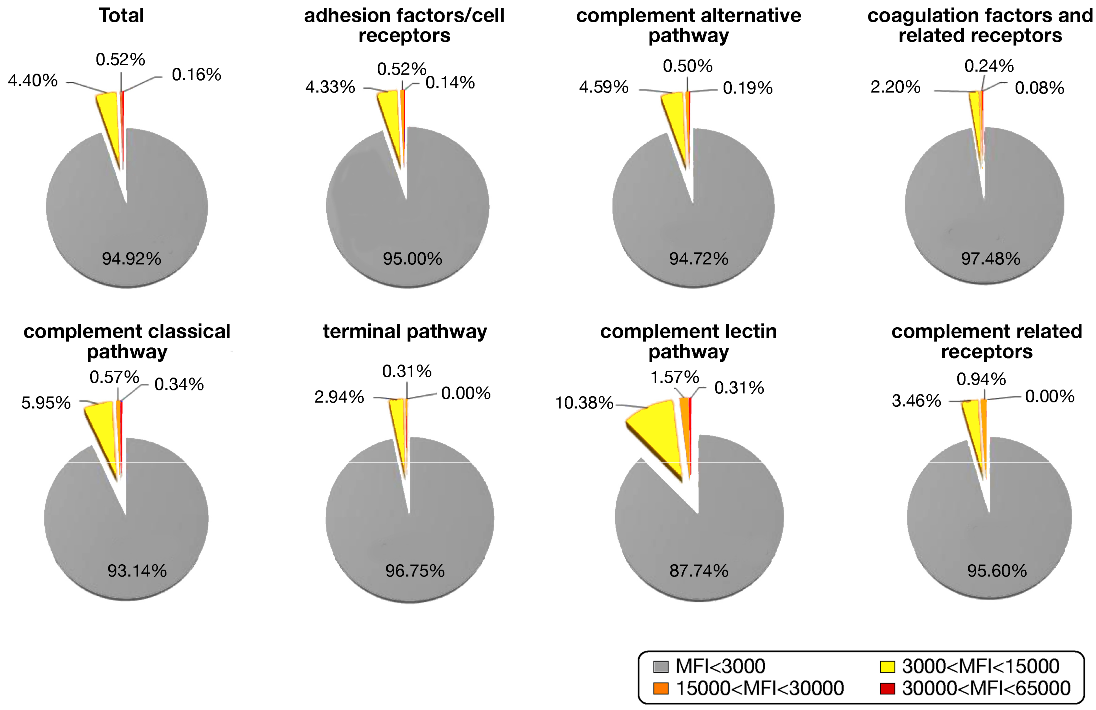

Supplementary Fig. S2 – FLIPr determination in bacterial supernatant. Western blot analysis using monoclonal antibodies against FLIPr on *S. aureus* supernatant (SN) Newman WT grown in TSB overnight until OD=11,7 (Stationary phase). The recombinant FLIPr (r-FLIPr) is used as positive control.

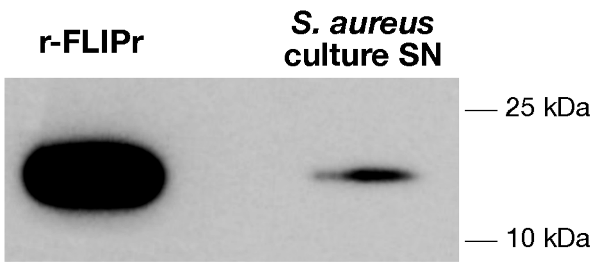

Supplementary Fig. S3 - DLS analysis of NadA, LOX-1 and the complex. Results are mean of 30 measurements. Measured hydrodynamic radius, sample polydispersity (%Pd) and calculated MW are shown in table.

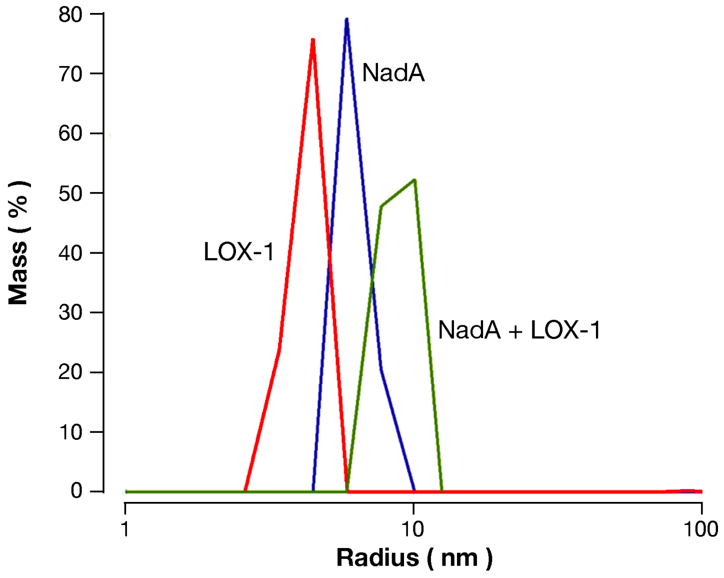

Sample	Radius (nm)	% Pd	MW (KDa)
LOX-1	4.206	10.8	97
NadA	6.217	11.9	242
Complex	8.928	13.4	565

Supplementary Fig. S4 - SEC-HPLC/MALLS measurements of the NadA$_{24\text{-}170}$ (left) and NadA$_{91\text{-}342}$(center) and NadA$_{24\text{-}89}$ (right). Light Scattering (LS - red), UV (green), and refractive index (RI - blue) signals are shown. Inset table and box shows respectively the calculated MW and the sample polydispersity.

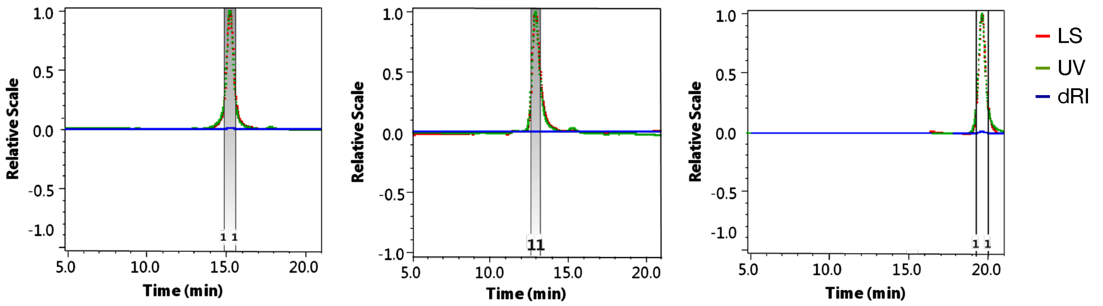

Sample	Absolute MW
NadAV3 A24-A170	52,34 kDa

Polydispersity
Mw/Mn 1.022 (±5.452%)
Mz/Mn 1.051(±9.259%)

Sample	Absolute MW
NadAV3 T91-G342	87,66 kDa

Polydispersity
Mw/Mn 1.022 (±10.943%)
Mz/Mn 1.056 (±18.683%)

Sample	Absolute MW
NadAV3 A24-V89	8,38 kDa

Polydispersity
Mw/Mn 1.020 (±9.105%)
Mz/Mn 1.044(±15.816%)

Supplementary Fig. S5 - Dot blot using mAbs 9F11, 1C9/A9 and 3C11/H7 on the NadA$_{24\text{-}170}$ and NadA$_{91\text{-}342}$ protein constructs and NadA. Exposure time: 10 min

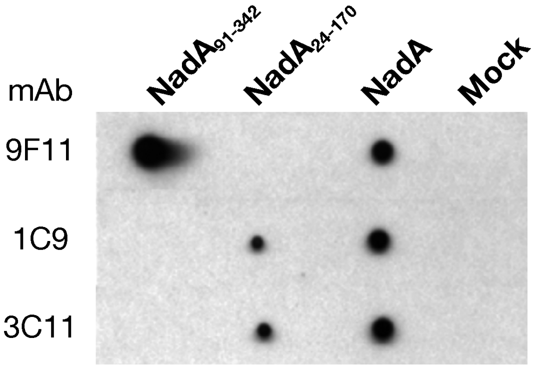

Supplementary Fig. S6 - Protein microarray validation. (A) Fluorescence imaging detected using anti-FLAG and anti mouse antibodies of the spotted amino terminal FLAG-tagged protein used as control. Dilutions and replicates of the curve are shown. BSA Cy3/Cy5 was used for coordinates referencing. **(B)** Plot of the FLAG-tagged protein dilutions MFI values over their concentration. Curves best fit in a sigmoid curve. Standard deviation for each concentration is shown and refers to MFI of 30 different slides. Red line represent background threshold.

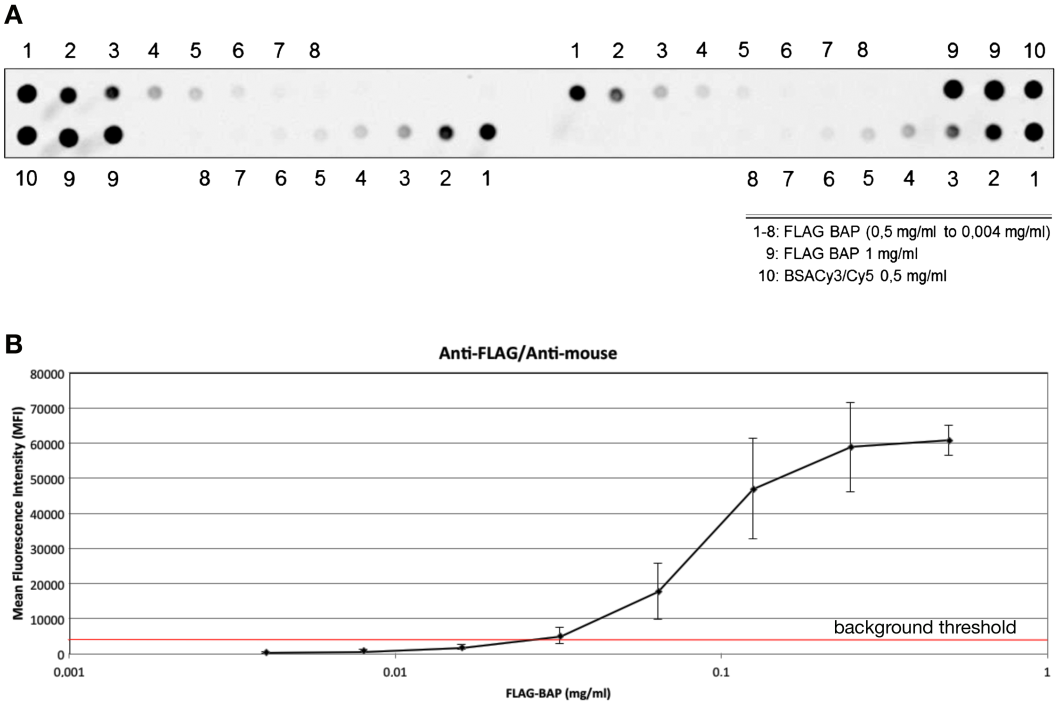

Supplementary Table S1 - **Name and function of the 75 human recombinant proteins** selected among the GNF library to be tested with the *S. aureus* protein microarray. The proteins are divided by function, based on the biological process they are involved in.

Function	Name	Gene name
adhesion / cell receptors / various	Adiponectin	ADIPOQ
	Anthrax toxin receptor 1	Antxr1
	Anthrax toxin receptor 2	Antxr2
	Basal cell adhesion molecule	BCAM
	Bone sialoprotein 2	IBSP
	Carcinoembryonic antigen related cell adhesion molecule 1	CEACAM1
	Cartilage oligomeric matrix protein	COMP
	Cell adhesion molecule 1	Cadm1
	Cell adhesion molecule 3	CADM3
	Cell surface glycoprotein MUC18	MCAM
	Endothelial cell selective adhesion molecule	ESAM
	Epithelial cell adhesion molecule	EPCAM
	Epithelial cell adhesion molecule precursor	EPCAMP
	Extracellular matrix protein 1	ECM1
	Extracellular matrix protein 2	ECM2
	Fibroleukin	FGL2
	Glucagon preproprotein	GCG
	Heat stable enterotoxin receptor	GUCY2C
	Integrin alpha L	ITGAL
	Integrin alpha M	ITGAM
	Integrin beta 2	ITGB2
	Intercellular adhesion molecule 1	ICAM1
	Intercellular adhesion molecule 2	ICAM2
	Intercellular adhesion molecule 3	ICAM3
	Intercellular adhesion molecule 4	ICAM4
	Intercellular adhesion molecule 5	ICAM5
	Matrix Gla protein	MGP
	Matrix remodeling associated protein 8	Mxra8
	Mucosal addressin cell adhesion molecule 1	Madcam1
	Neural cell adhesion molecule 1	NCAM1
	Neural cell adhesion molecule 2	NCAM2
	Single immunoglob tollinterleu 1 receptor (TIR)	SIGIRR
	Spondin 1	SPON1
	Toll like receptor 2	TLR2
	Vitronectin	VTN
complement alternative pathway	CD46 molecule complement regulatory protein	CD46
	Complement factor B	CFB
	Complement factor D (adipsin)	CFD
	Complement Factor H	CFH
	Complement factor H related 1	CFHR1
	Complement factor H related 2	CFHR2
	Complement factor H related 4	CFHR4
	Complement factor H related 5	CFHR5
	Complement factor I	CFI
	Complement factor properdin	CFP
coagulation factors and related receptors	Fibrinogen beta chain	FGB
	Fibrinogen gamma chain	FGG
	Fibrinogen like protein 1	FGL1
	Platelet endot cell adhesion molecule precursor	PECAM1
	Platelet endothelial cell adhesion molecule	PECAM1
	Von W factor C domain protein like 2	VWA2
	Von Will factor A domain containing protein 2	VWCE
	Von Willebrand factor C and EGF domain containing protein	VWC2L

complement classical pathway	Complement component 1 q subcomponent A chain	C1QA
	Complement component 1 q subcomponent B chain	C1QB
	Complement component 1 q subcomponent C chain	C1QC
	Complement component 1 q subcomponent like 1	C1QL1
	Complement component 1 q subcomponent like 2	C1QL2
	Complement component 1 q subcomponent like 4	C1QL4
	Complement component 1 r subcomponent	C1R
	Complement component 1 r subcomponent like	C1RL
	Complement component 1 s subcomponent	C1S
	Complement component 2 (within H 2S)	C2
	Complement component 4 binding protein, beta	C4BPB
complement terminal pathway	Complement component 6	C6
	Complement component 7	C7
	Complement component 8 alpha polypeptide	C8A
	Complement component 8 beta polypeptide	C8B
	Complement component 8 gamma polypeptide	C8G
	Complement factor 9	C9
complement lectin pathway	Ficolin 1	FCN1
	Ficolin 2	FCN2
	Ficolin 3	FCN3
complement related receptors	Compl comp (3d Epstein Barr virus) receptor 2	CR1L
	Complement component (3b4b) receptor 1 like	CR2

Supplementary Table S2 - Common name, locus tag, gene product and length and localization of the *S. aureus* recombinant proteins printed on the microarray slides. *Neisseria meningitidis* factor H binding protein (fHbp) used as positive control is also present in the list.

Common name	Locus tag	gene product	gene length	localization
	gna1870	factor h binding protein	819	Outer membrane
	gna1870	factor h binding protein	819	Outer membrane
	gna1870	factor h binding protein	819	Outer membrane
	SAOUHSC_00051	1-phosphatidylinositol phosphodiesterase precursor, putative	328	Extracellular
	SAOUHSC_00052	putative lipoprotein	256	Unknown
	SAOUHSC_00053	putative lipoprotein	256	Unknown
	SAOUHSC_00054	staphylococcal tandem lipoprotein	256	Unknown
	SAOUHSC_00055	staphylococcal tandem lipoprotein	255	Unknown
spa	SAOUHSC_00069	protein A spA	516	Cell wall
sasD	SAOUHSC_00094	SasD protein SAOUHSC_00094	199	Cell wall
	SAOUHSC_00106	hypothetical protein , leader	514	Unknown
	SAOUHSC_00107	5' nucleotidase family protein	511	Extracellular
isdI	SAOUHSC_00130	heme-degrading monooxygenase IsdI	108	Unknown
	SAOUHSC_00170	extracellular solute-binding protein, RGD containing lipoprotein	591	Unknown
	SAOUHSC_00171	gamma-glutamyltranspeptidase, putative	668	Extracellular
	SAOUHSC_00172	hypothetical protein SAOUHSC_00172	257	Unknown
	SAOUHSC_00174	M23/M37 peptidase domain protein	192	Extracellular
	SAOUHSC_00176	bacterial extracellular solute-binding protein, putative	423	Unknown
	SAOUHSC_00186	lipoprotein, putative	322	Unknown
coA	SAOUHSC_00192	coagulase Coa	636	Extracellular
	SAOUHSC_00201	putative extracellular solute-binding protein	470	Cell wall
	SAOUHSC_00248	peptidoglycan hydrolase, putative	316	Extracellular
	SAOUHSC_00256	secretory antigen SsaA-like protein	297	Unknown
esxA	SAOUHSC_00257	hypothetical protein SAOUHSC_00257	97	Unknown
esxB	SAOUHSC_00265	hypothetical protein SAOUHSC_00265	104	Cytoplasmic
	SAOUHSC_00279	putative lipoprotein	124	Cytoplasmic
	SAOUHSC_00300	lipase precursor	690	Extracellular
	SAOUHSC_00354	staphylococcal enterotoxix, putative	203	Unknown
	SAOUHSC_00356	putative lipoprotein	190	Unknown
	SAOUHSC_00362	putative lipoprotein	208	Unknown
	SAOUHSC_00365	alkyl hydroperoxide reductase	189	Cytoplasmic
	SAOUHSC_00383	superantigen-like protein	226	Unknown
	SAOUHSC_00384	superantigen-like protein	231	Unknown
	SAOUHSC_00386	superantigen-like protein	356	Extracellular
	SAOUHSC_00389	superantigen-like protein	308	Extracellular
	SAOUHSC_00390	superantigen-like protein 5	234	Extracellular
	SAOUHSC_00391	superantigen-like protein	231	Extracellular
	SAOUHSC_00392	superantigen-like protein 7	231	Extracellular
	SAOUHSC_00393	superantigen-like protein	232	Extracellular
	SAOUHSC_00394	superantigen-like protein	232	Extracellular
	SAOUHSC_00395	superantigen-like protein	227	Extracellular

	SAOUHSC_00399	superantigen-like protein	225	Extracellular
	SAOUHSC_00400	putative surface protein	502	Unknown
	SAOUHSC_00404	putative lipoprotein	261	Unknown
	SAOUHSC_00427	autolysin precursor, putative	334	Extracellular
sdrC	SAOUHSC_00544	sdrC protein, putative	995	Cell wall
sdrD	SAOUHSC_00545	sdrD protein, putative	1349	Cell wall
	SAOUHSC_00634	ABC transporter, substrate-binding protein, putative \lipoprotein	312	Unknown
	SAOUHSC_00661	probable lipase	347	Unknown
	SAOUHSC_00671	secretory antigen SsaA-like protein	265	Extracellular
	SAOUHSC_00685	putative lipoprotein	131	Unknown
	SAOUHSC_00717	lipoprotein	146	Unknown
	SAOUHSC_00728	Predicted membrane-associated, metal-dependent hydrolase	646	Cytoplasmic/Membrane
	SAOUHSC_00749	Siderophore binding protein FatB	342	Unknown
	SAOUHSC_00754	ferrichrome binding protein	292	Unknown
	SAOUHSC_00773	immunogenic secreted precursor-like protein (truncated)	279	Unknown
	SAOUHSC_00808	putative lipoprotein	242	Unknown
	SAOUHSC_00814	truncated secreted von Willebrand factor-binding protein (coagulase) VWbp, putative	450	Extracellular
Emp	SAOUHSC_00816	extracellular matrix and plasma binding protein, putative	340	Unknown
	SAOUHSC_00817	hypothetical protein SAOUHSC_00817 \ von willebrand truncated	156	Unknown
	SAOUHSC_00860	5-nucleotidase family protein	439	Extracellular
	SAOUHSC_00872	extramembranal protein	391	Cytoplasmic
	SAOUHSC_00987	cysteine protease precursor, putative	393	Extracellular
	SAOUHSC_00988	glutamyl endopeptidase precursor, putative	336	Extracellular
	SAOUHSC_00998	fmt protein, putative	397	Extracellular
	SAOUHSC_01005	chitinase	105	Unknown
	SAOUHSC_01039	putative lipoprotein	208	Unknown
isdB	SAOUHSC_01079	neurofilament protein isdB	645	Cell wall
isdA	SAOUHSC_01081	IsdA protein	350	Cell wall
IsdC	SAOUHSC_01082	hypothetical protein SAOUHSC_01082 isdC	227	Cellwall
	SAOUHSC_01084	hypothetical iron-regulated protein, leader isdD	358	Unknown
	SAOUHSC_01085	iron ABC transporter, iron -binding protein IsdE	282	Cytoplasmic
	SAOUHSC_01088	NPQTN specific sortase B	244	Unknown
isdG	SAOUHSC_01089	heme-degrading monooxygenase IsdG	107	Cytoplasmic
	SAOUHSC_01110	fibrinogen-binding protein-related	109	Unknown
FLIPr	SAOUHSC_01112	formyl peptide receptor-like 1 inhibitory protein	133	Unknown
efb	SAOUHSC_01114	fibrinogen-binding protein	165	Extracellular
	SAOUHSC_01115	scin paralogue	116	Unknown
hla	SAOUHSC_01121	alpha-hemolysin precursor	319	Extracellular
	SAOUHSC_01124	superantigen-like protein	238	Extracellular
	SAOUHSC_01125	superantigen-like protein	241	Extracellular
	SAOUHSC_01150	cell division protein FtsZ	390	Cytoplasmic
	SAOUHSC_01180	putative lipoprotein	317	Unknown
	SAOUHSC_01256	insulysin, peptidase family M16	428	Unknown
nuc	SAOUHSC_01316	thermonuclease precursor	177	Extracellular
	SAOUHSC_01317	hypothetical protein leader?	284	Unknown
ebpS	SAOUHSC_01501	elastin binding protein EbpS	486	Cell wall

	SAOUHSC_01508	putative lipoprotein	304	Unknown
	SAOUHSC_01627	putative lipoprotein	193	Unknown
	SAOUHSC_01920	putative lipoprotein	208	Unknown
	SAOUHSC_01941	serine protease SplB	240	Extracellular
	SAOUHSC_01949	intracellular serine protease, putative	457	Extracellular
	SAOUHSC_01972	protein export protein PrsA, putative	320	Unknown
	SAOUHSC_02127	staphopain thiol proteinase	388	Extracellular
	SAOUHSC_02147	hypothetical protein, putative leader	280	Unknown
eap	SAOUHSC_02161	MHC class II analog protein	584	Unknown
	SAOUHSC_02167	hypothetical protein SAOUHSC_02167	116	Unknown
	SAOUHSC_02169	chemotaxis-inhibiting protein CHIPS	149	Unknown
	SAOUHSC_02240	truncated beta-hemolysin	274	Extracellular
lukF	SAOUHSC_02241	Leukocidin/Hemolysin toxin family LukF	338	Extracellular
	SAOUHSC_02246	ferric hydroxamate receptor 1	303	Unknown
	SAOUHSC_02257	srdH family protein	419	Cell wall
	SAOUHSC_02333	Probable transglycosylase isaA precursor	231	Extracellular
	SAOUHSC_02463	hyaluronate lyase	807	Extracellular
	SAOUHSC_02554	ferrichrome-binding protein TroA-like -FhuD2?	302	Unknown
	SAOUHSC_02576	secretory antigen precursor SsaA, putative	166	Unknown
	SAOUHSC_02706	immunoglobulin G-binding protein Sbi, putative	436	Unknown
	SAOUHSC_02708	gamma-hemolysin h-gamma-ii subunit, putative	309	Extracellular
	SAOUHSC_02767	peptide ABC transporter, peptide-binding protein, putative	532	Cell wall
	SAOUHSC_02783	hypothetical protein, putative leader	264	Unknown
FnBA	SAOUHSC_02803	fibronectin-binding protein A precursor FnBPA	990	Cell wall
	SAOUHSC_02887	immunodominant antigen A, putative	233	Extracellular
clfB	SAOUHSC_02963	clumping factor B, putative	877	Cell wall
	SAOUHSC_02979	N-acetylmuramoyl-L-alanine amidase	619	Extracellular
sasF	SAOUHSC_02982	sasF protein	635	Cell wall
	SAOUHSC_03006	lipase	680	Extracellular
NW_sdrE	NWMN_0525	Serine-aspartate repeat-containing protein E	3498	Unknown

Supplementary Table S3 - Detailed microarray analysis results divided based on human protein classes. Protein classes are shown in columns; colors used are the same as in figure 2A. Ficolin-2 was not considered in the analysis.

	adhesion factors/cell receptors/ others	complement alternative pathway	coagulation factors and related receptors	complement classical pathway	complement cascade	complement lectin pathway	complement related receptors	total
Proteins	35	10	8	11	6	2	2	74
total n of combinations	5565	1590	1272	1749	954	318	318	11766
negative combinations	5287	1506	1240	1629	923	279	304	11168
combinations between 3K 15K MFI	241	73	28	104	28	33	11	518
combinations between 15K 30K MFI	29	8	3	10	3	5	3	61
combinations between 30K 65K MFI	8	3	1	6	0	1	0	19

Supplementary Table S4 - List of the 19 interactions between *S. aureus* and human proteins with an MFI value from 30000 to saturation. Locus tag is referred to the *Staphylococcus aureus* NCTC 8325 strain. Gene name is indicated for human proteins**.**

S. aureus protein	Locus Tag	MFI value	Human protein	Gene name	Human protein tag
LytM	SAOUHSC_00248	59922	Complement factor properdin	CFP	CFlagHis
fHbp v1	gna1870	57788	Complement Factor H	CFH	CFlagHis
fHbp v3	gna1870	57552	Complement Factor H	CFH	CFlagHis
FLIPr	SAOUHSC_01112	56363	Intercellular adhesion molecule 5	ICAM5	NFcFlagHis
spA	SAOUHSC_00069	53285	Complement component 4 binding protein, beta	C4BPB	CFcFlagHis
FLIPr	SAOUHSC_01112	52776	Heat stable enterotoxin receptor	GUCY2C	NFcFlagHis
FLIPr	SAOUHSC_01112	47822	Complement component 1 q subcomponent like 4	C1QL4	CFlagHis
spA	SAOUHSC_00069	46199	Complement component 2 (within H 2S)	C2	NFcFlagHis
LytM	SAOUHSC_00248	42215	Adiponectin	ADIPOQ	CFlagHis
FLIPr	SAOUHSC_01112	41675	Matrix remodeling associated protein 8	Mxra8	CFcFlagHis
FLIPr	SAOUHSC_01112	38610	Complement component 1 q subcomponent B	C1QB	CFlagHis
FLIPr	SAOUHSC_01112	37018	Bone sialoprotein 2	IBSP	NFcFlagHis
spA	SAOUHSC_00069	35544	Complement component 1 r subcomponent	C1R	NFcFlagHis
spA	SAOUHSC_00069	34383	Fibrinogen like protein 1	FGL1	CFcFlagHis
Csa1D	SAOUHSC_00055	34016	Ficolin 1	FCN1	CFcFlagHis
spA	SAOUHSC_00069	33699	Matrix Gla protein	MGP	NFcFlagHis
spA	SAOUHSC_00069	33570	Extracellular matrix protein 2	ECM2	NFcFlagHis
LytM	SAOUHSC_00248	32863	Complement component 1 q subcomponent like 4	C1QL4	CFlagHis
LytM	SAOUHSC_00248	31751	Matrix remodeling associated protein 8	Mxra8	CFcFlagHis
